# Piwi-interacting RNAs and PIWI genes as novel prognostic markers for breast cancer

**DOI:** 10.18632/oncotarget.9272

**Published:** 2016-05-10

**Authors:** Preethi Krishnan, Sunita Ghosh, Kathryn Graham, John R. Mackey, Olga Kovalchuk, Sambasivarao Damaraju

**Affiliations:** ^1^ Department of Laboratory Medicine and Pathology, University of Alberta, Edmonton, Alberta, Canada; ^2^ Department of Oncology, University of Alberta, Edmonton, Alberta, Canada; ^3^ Cross Cancer Institute, Alberta Health Services, Edmonton, Alberta, Canada; ^4^ Department of Biological Sciences, University of Lethbridge, Lethbridge, Alberta, Canada

**Keywords:** piRNA, PIWI, breast cancer, prognostic marker, TCGA

## Abstract

Piwi-interacting RNAs (piRNAs), whose role in germline maintenance has been established, are now also being classified as post-transcriptional regulators of gene expression in somatic cells. PIWI proteins, central to piRNA biogenesis, have been identified as genetic and epigenetic regulators of gene expression. piRNAs/PIWIs have emerged as potential biomarkers for cancer but their relevance to breast cancer has not been comprehensively studied. piRNAs and mRNAs were profiled from normal and breast tumor tissues using next generation sequencing and Agilent platforms, respectively. Gene targets for differentially expressed piRNAs were identified from mRNA expression dataset. piRNAs and PIWI genes were independently assessed for their prognostic significance (outcomes: Overall Survival, OS and Recurrence Free Survival, RFS). We discovered eight piRNAs as novel independent prognostic markers and their association with OS was confirmed in an external dataset (The Cancer Genome Atlas). Further, PIWIL3 and PIWIL4 genes showed prognostic relevance. 306 gene targets exhibited reciprocal relationship with piRNA expression. Cancer cell pathways such as apoptosis and cell signaling were the key Gene Ontology terms associated with the regulated gene targets. Overall, we have captured the entire cascade of events in a dysregulated piRNA pathway and have identified novel markers for breast cancer prognostication.

## INTRODUCTION

Piwi-interacting RNAs (piRNAs, 24 – 32 nt in length) belong to a class of small regulatory RNAs that include microRNAs (miRNAs) and small interfering RNAs (siRNAs) [1]. Mature forms of these RNAs associate with biogenesis pathway proteins such as Argonaute (AGO) class of proteins: miRNAs and siRNAs with AGO proteins and piRNAs with PIWI proteins [2–5] to guide target specific gene regulation [6, 7]. Gene regulation exerts control at transcriptional and post-transcriptional levels and piRNAs, in association with PIWI proteins, are involved in both levels [8, 9]. For a long time, the only roles of PIWI proteins were believed to be in the regulation of transposons and [10] in the maintenance and development of germinal stem cells [11]; however, the functions of piRNAs and PIWI proteins as epigenetic regulators have started to emerge [1, 12]. It is now known that PIWI proteins, which are guided by piRNAs bind to specific targets (based on sequence specific complementarity) and recruit chromatin modifiers to enable transcriptional repression [13]. Apart from this, a direct association between the piRNA–PIWI protein complex and stem cell development and maintenance has been established [14]. Cancer stem cells form a critical fraction of a tumor mass, are required for incessant cell proliferation, and may underlie resistance to drugs and radiation; accordingly, cancer stem cells are believed to contribute to tumor recurrence [15, 16]. The role of the piRNA–PIWI protein complex in post-transcriptional gene regulation is also slowly garnering attention. Although the exact mechanism remains elusive, investigators initially have reported the sequence specific complementary binding of a piRNA to a target messenger RNA (mRNA) at the 3′ untranslated region (UTR) and subsequent gene regulation, in a manner similar to that of miRNAs [17–19]. It is increasingly being recognized that the sequence based complementarity may not be restricted to 3′ UTR and may expand to 5′UTR, the coding sequence or even the introns [20]. Given the diverse functions of piRNAs and PIWI proteins, it is evident that these molecules may also contribute to tumorigenesis [9].

Human homologues of PIWI proteins (originally described as P-element induced wimpy testis in Drosophila) identified thus far are PIWIL1 (HIWI), PIWIL2 (HILI), PIWIL3 and PIWIL4 (HIWI2) [21]. Although the expression of PIWI proteins in somatic tissues has been known since 1998, our major understanding of these molecules stem from germ cells. Only recently, have researchers demonstrated their possible involvement in tumorigenesis [9]. Aberrant expressions of these genes and proteins in malignancy have been associated with hallmarks of cancer and have also shown promise as potential prognostic and diagnostic markers for different cancer types [22]. In this regard, the differential expression of piRNAs and therefore their oncogenic or tumor suppressor roles have also been observed in various cancer types [19, 20], and a few studies have highlighted their association with clinicopathological factors [23]. An even smaller number of studies have reported the relevance of piRNAs as prognostic/diagnostic markers [24–26]; however, the study designs of the majority of these studies are limited to candidate piRNA molecules or are challenged with limited sample sizes.

Given the current knowledge that piRNAs and PIWI genes (i) are abundantly expressed in somatic tissues, (ii) are potential biomarkers for cancer and (iii) are involved in gene regulation and in normal developmental processes, extensive profiling and characterization studies are needed to understand the contribution of these molecules to tumorigenesis. The contribution of both piRNAs and PIWI genes to breast cancer has not been comprehensively studied and is the focus of this report. We hypothesized that varying levels of piRNAs and their upstream biogenesis pathway (PIWI) genes contribute to breast tumorigenesis and act as prognostic markers for breast cancer. Our specific objectives were (i) to identify differentially expressed piRNAs and PIWI gene transcripts (mRNAs) (hereafter referred to as PIWI genes) in breast tumor tissues relative to normal (reduction mammoplasty) breast tissues, (ii) to identify piRNAs and PIWI genes as prognostic markers (outcomes: overall survival, OS and recurrence free survival, RFS) and (iii) to identify complementary gene (mRNA) targets at the 3′ UTR for the piRNAs associated with breast cancer prognosis.

## RESULTS

### piRNAs are expressed in breast tissues

**Figure 1:**
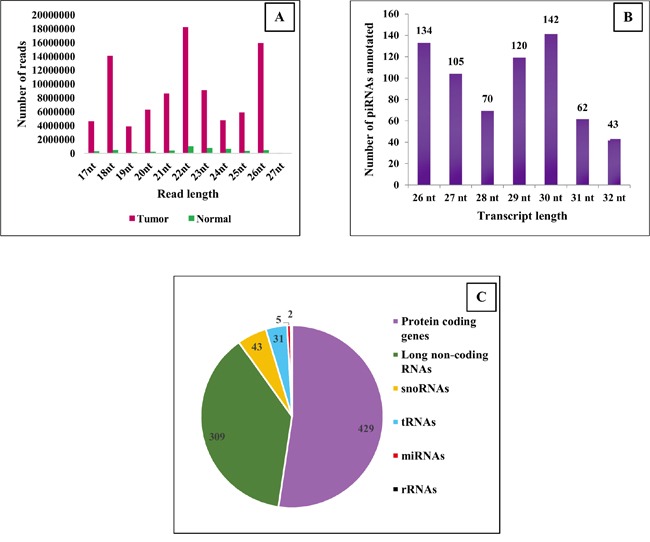
piRNAs in breast tissues **A**: The above histogram corresponds to the number of reads mapping to different read length sizes. **B**: The numbers of annotated piRNAs identified under different transcript lengths are indicated. **C**: Pie-chart shows the number of piRNAs mapping to different classes of genes. ~61% of the piRNAs profiled in our study map to exons and introns of protein coding genes.

The next generation sequencing (NGS) experiment generated approximately 10 million reads from normal tissues and about 165 million reads from tumor tissues. A good 50–60% of the reads were retained in both the tissues after trimming the adapters, and about 85% of the reads (88 million reads in total from both tissue types) aligned to the human genome (hg19). Among the reads that aligned, 4,207,022 were classified as piRNAs, which annotated to 676 individual piRNAs. The sequencing protocol followed was 36 cycles single end protocol. Of the 36 nucleotides, 7 belonged to the index sequence and reads ranging from 17 – 27 nucleotides were retained (Figure 1A), representing the RNA species from miRNA, piRNA, tRNA etc. The actual lengths of the piRNAs range from 24-32 nt (as annotated in piRNA database [27]). The 676 piRNAs identified were in the range 26-32 nt (Figure 1B). We recognize that not all of piRNAs on the genome were captured due to limitations of the library construction. This is unlikely to have affected the results and interpretations of the current study. We observed similar patterns of distributions for piRNA reads from the TCGA data set, also generated from the 36 cycle single end protocol (data not shown).

piRNAs have predominantly been studied in germline cells and, have only recently been reported in somatic tissues. In the germline, they are most commonly seen as clusters; while in the somatic tissues, they have been observed to be mapping to intronic and exonic regions of several protein-coding genes [26]. We confirmed these findings in our profiling experiments for piRNA expression from somatic breast tissues. Of the 676 piRNAs profiled, 429 mapped to exons and introns of known protein coding genes, and 309 mapped to exons and introns of long non-coding RNAs. A few of the piRNAs also mapped to other non-coding RNA classes such as miRNAs, tRNAs and snoRNAs (Figure 1C, Supplementary Table S1).

### piRNAs are potential independent prognostic markers for breast cancer

#### Case–control (CC) method

**Figure 2:**
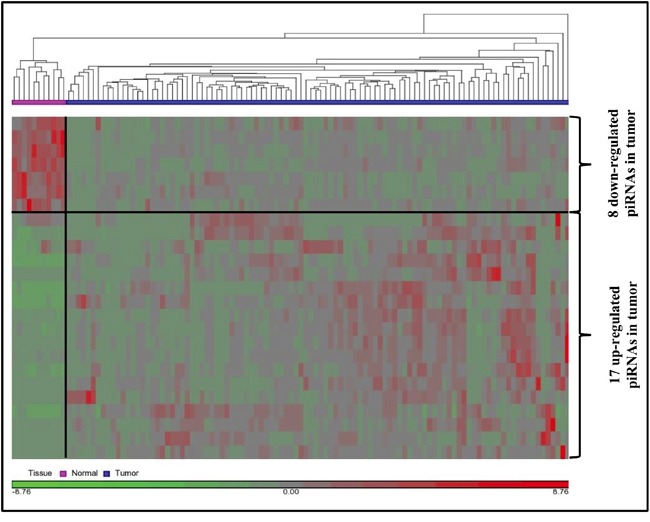
Differential expression of piRNAs 25 differentially expressed piRNAs were used for unsupervised hierarchical clustering using average linkage method for linkage analysis and Euclidean distance measure.

The raw data was normalized using the RPKM method and was adjusted for any potential batch effects (Supplementary Figure S1). One sample was identified as a potential outlier and was removed, leaving 102 breast tumor tissues and 11 normal tissues for further analysis. Out of the 676 piRNAs profiled, 42 were retained after filtering for read counts and 25 were identified as differentially expressed (DE). 17 piRNAs were up-regulated and eight were down-regulated with fold change (FC) > 2 and False Discovery Rate cut–off 0.05 (Figure 2, Supplementary Table S2). Raw counts of the 676 piRNAs and normalized counts (after adjusting for batch effects) of 676 and 42 filtered piRNAs are provided in Supplementary Table S3.

**Figure 3:**
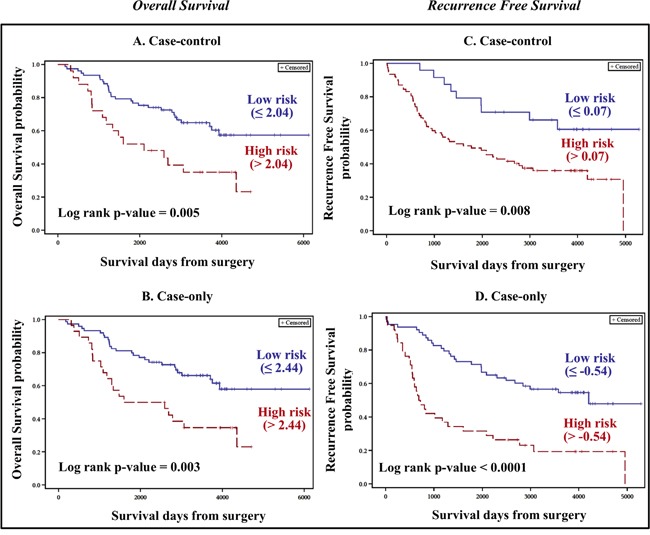
Kaplan-Meier plots for constructed risk scores (Discovery cohort) Risk scores were constructed using piRNAs significant in univariate Cox analysis with permuted p-value ≤ 0.1. For both case-control and case-only paradigms in the discovery cohort, samples were dichotomized into low and high risk groups based on ROC estimation of optimal cut-off point (indicated in parenthesis). In all the comparisons, patients belonging to high risk group were associated with shorter survival periods (OS and RFS), with log-rank p value < 0.05. **A.** (case–control) and **B.** (case–only) represent Kaplan Meier plots for Overall survival. **C.** (case–control) and **D.** (case–only) represent Kaplan Meier plots for Recurrence free survival corresponding to discovery cohort.

Of the 25 DE piRNAs, three piRNAs each were significant (permuted p value < 0.1) in the univariate analysis for OS and RFS (Supplementary Table S4) and were used to construct the individual risk scores. Two piRNAs (i.e. hsa_piR_009051 and hsa_piR_021032) were significant for both OS and RFS. The receiver operating characteristics (ROC) curve estimated cut-off points for OS and RFS were 2.04 and 0.07, respectively, dichotomizing the patients into low–risk (≤ 2.04 for OS and ≤ 0.07 for RFS) and high–risk (> 2.04 for OS and > 0.07 for RFS) groups. The risk scores were found to be significant after adjusting for tumor stage and age at diagnosis for OS and tumor stage for RFS (Table 1). Patients belonging to the high–risk group were associated with poor OS (Figure 3A) and RFS (Figure 3C).

**Table 1:** Univariate and multivariate results of piRNAs identified in Case–control method (Discovery cohort)

Parameter	Overall Survival				Recurrence Free Survival			
	Univariate analysis		Multivariate analysis		Univariate analysis		Multivariate analysis	
	HR (95% CI)	p-value	HR (95% CI)	p-value	HR (95% CI)	p-value	HR (95% CI)	p-value
**Risk score**	2.31 (1.27 – 4.22)	0.01	2.29 (1.24 – 4.27)	0.01	2.53 (1.25 – 5.16)	0.01	2.79 (1.36 – 5.69)	0.005
**Tumor stage**	0.40 (0.21 – 0.78)	0.01	0.42 (0.21 – 0.84)	0.02	0.38 (0.20 – 0.71)	0.003	0.34 (0.18 – 0.63)	0.001
**Tumor grade**	2.01 (1.04 – 3.89)	0.04			1.58 (0.92 – 2.74)	0.1		
**Age at diagnosis**	1.06 (1.02 – 1.09)	0.001	1.04 (1.01 – 1.08)	0.01	1.02 (0.99 – 1.05)	0.21		
**TNBC status**	0.99br (1.16 – 3.29)	0.98			0.84 (0.45 – 1.55)	0.58		

HR = Hazards ratio; CI = Confidence interval

**Table 1.** Univariate and multivariate Cox analysis results for OS (left panel) and RFS (right panel) in case–control approach is represented. Patients belonging to high–risk group were associated with poor prognosis (HR > 1) and the risk score showed promise as potential independent prognostic factor (p < 0.05).

#### Case–only (CO) method

665 piRNAs were expressed with at least one read count in any one of the tumor samples. 53 piRNAs were retained with ≥ 10 read counts and expressed in at least 90% of the tumor samples. The raw data was adjusted for batch effects. The raw data for all 665 piRNAs and the batch effects adjusted normalized counts of 665 and 53 filtered piRNAs are provided in Supplementary Table S3. Four and six piRNAs (from the 53 filtered piRNAs) were significant in the univariate analysis for OS and RFS (Supplementary Table S4) with a permuted p-value ≤ 0.1. The risk scores were constructed using the four and six piRNAs for OS and RFS, respectively. The ROC based estimation of the cut–off point dichotomized the patients into two groups: low–risk (≤ 2.44 for OS and ≤ −0.54 for RFS) and high–risk (> 2.44 for OS and > −0.54 for RFS). For both outcomes, (i) the risk score showed p–value significance in the univariate and multivariate analyses (Table 2) after adjusting for potential confounders (tumor grade and age at diagnosis for OS and tumor stage for RFS) and (ii) the high–risk group patients showed poor OS (Figure 3B) and RFS (Figure 3D).

**Table 2:** Univariate and multivariate results of piRNAs identified in case–only method (Discovery cohort)

Parameter	Overall Survival				Recurrence free Survival			
	Univariate analysis		Multivariate analysis		Univariate analysis		Multivariate analysis	
	HR (95% CI)	p-value	HR (95% CI)	p-value	HR (95% CI)	p-value	HR (95% CI)	p-value
**Risk score**	2.36 (1.31 – 4.26)	0.004	2.09 (1.15 – 3.79)	0.02	3.08 (1.84 – 5.16)	<0.0001	3.07 (1.84 – 5.14)	<0.0001
**Tumor stage**	0.40 (0.21 – 0.78)	0.01			0.38 (0.20 – 0.71)	0.003	0.39 (0.21 – 0.72)	0.003
**Tumor grade**	2.01 (1.04 – 3.89)	0.04	2.01 (1.03 – 3.92)	0.04	1.58 (0.92 – 2.74)	0.1		
**Age at diagnosis**	1.06 (1.02 – 1.09)	0.001	1.06 (1.02 – 1.09)	0.001	1.02 (0.99 – 1.05)	0.21		
**TNBC status**	0.99 (0.50 – 1.95)	0.98			0.84 (0.45 – 1.55)	0.58		

HR = Hazards ratio; CI = Confidence interval

**Table 2.** Univariate and multivariate Cox analysis results for OS (left panel) and RFS (right panel) in case–only approach are represented. Patients belonging to high–risk group were associated with poor prognosis (HR > 1) and the risk score showed promise as potential independent prognostic factor (p < 0.05).

### The risk score for OS was significant in the external validation dataset

**Figure 4:**
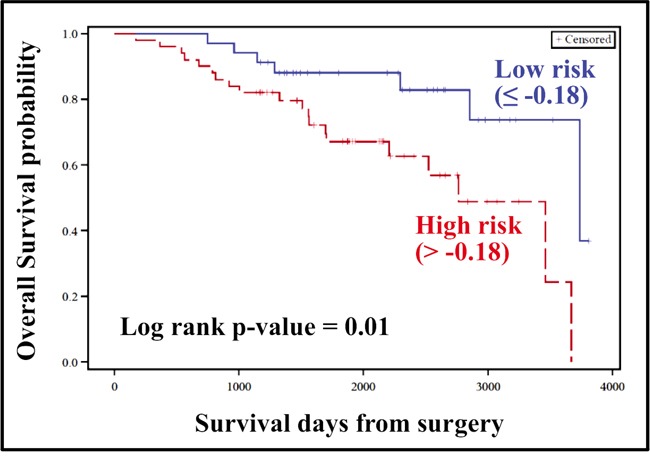
Kaplan–Meier plot for constructed risk score (External validation/TCGA dataset) Risk score for Overall survival was constructed using piRNAs significant in univariate Cox analysis with permuted p-value ≤ 0.1, as identified in the discovery cohort. Further, samples were dichotomized into low and high risk groups based on ROC estimation of optimal cut-off point (indicated in parenthesis). Patients belonging to high risk group were associated with shorter survival period (OS), with log-rank p value < 0.05, confirming the results obtained in the discovery cohort.

Batch–adjusted normalized counts of the four piRNAs (significant for OS in the discovery cohort) were extracted from the 84 samples in The Cancer Genome Atlas (TCGA) dataset. A risk score was constructed for OS, and the ROC based estimation of the cut–off point dichotomized the samples into low–risk (≤ −0.18) and high–risk (> −0.18) groups. Similar to the results obtained in the discovery cohort, the risk score showed promise as potential independent prognostic factor (Table 3), and patients in the high–risk group were significantly associated with poor OS (Figure 4; p<0.01).

**Table 3:** Univariate and multivariate results for Overall Survival (External validation/TCGA dataset)

Parameter	Overall Survival			
	Univariate analysis		Multivariate analysis	
	HR (95% CI)	p-value	HR (95% CI)	p-value
**Risk score**	3.02 (1.21 – 7.59)	0.02	3.22 (1.22 – 8.52)	0.02
**Tumor stage**	0.32 (0.13 – 0.78)	0.01	0.34 (0.14 – 0.88)	0.03
**Age at diagnosis**	1.03 (1.003 – 1.06)	0.03	1.04 (1.01 – 1.07)	0.006
**TNBC status**	0.63 (0.19 – 2.12)	0.46		

HR = Hazards ratio; CI = confidence interval

**Table 3.** Risk score constructed using four piRNAs (identified as significant for OS in discovery cohort) was adjusted for tumor stage and age at diagnosis and was found to be significant with p < 0.05 in TCGA dataset (external validation set).

### PIWI genes are promising prognostic markers for breast cancer

**Figure 5:**
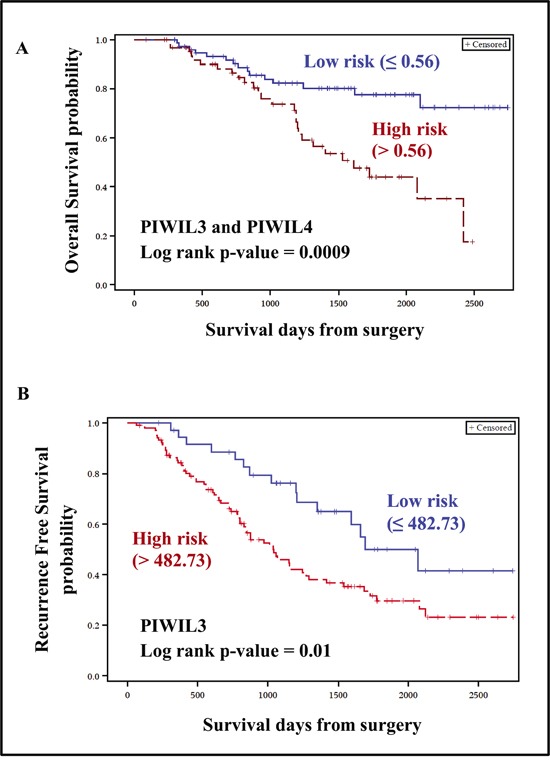
Kaplan-Meier plots for PIWI genes PIWIL3 and PIWIL4 genes were significant for OS and were used for constructing a risk score, whereas PIWIL3 alone was significant for RFS. Patients were dichotomized into low and high-risk groups based on ROC estimated cut-off point (indicated in parenthesis). Patients belonging to high-risk group were associated with poor OS (A) and RFS (B).

All four human homologues of PIWI genes were expressed in our in-house breast cancer gene expression dataset. Comparison with normal breast tissues revealed that two genes (PIWIL1 and PIWIL3) were up-regulated and the remaining two (PIWIL2 and PIWIL4) were down-regulated in tumor tissues (Table 4). The up-regulated PIWI genes did not show statistical significance between normal and breast tumor tissues. Nevertheless, we confirmed the expression of PIWI genes in breast (somatic) tissues. Since these proteins are involved in piRNA biogenesis, an aberrant expression of these genes in breast cancer may contribute to abnormal expression of piRNAs. As we had identified the prognostic significance of piRNAs, we hypothesized that genes coding for PIWI proteins may also be involved in breast cancer prognosis. Of the four PIWI genes, only PIWIL3 and PIWIL4 genes were significant in the univariate analysis for OS and were used to construct a risk score. Similar to the piRNA analysis, ROC was used to estimate the optimal cut–off point for dichotomization of patients into low–risk (≤ 0.56) and high–risk (> 0.56) groups. The risk score was significant for OS after adjusting for age at diagnosis and Triple Negative Breast Cancer (TNBC) status (Table 5). In the case of RFS, PIWIL3 gene was found to be significant. The potential of PIWIL3 gene as an independent prognostic marker was confirmed in the multivariate analysis (Table 5). For both OS (Figure 5A) and RFS (Figure 5B), patients belonging to the high–risk group were found to have shorter survival.

**Table 4:** Differential expression of PIWI genes

PIWI gene	Fold change	Direction of expression	p-value
PIWIL1	1.56	Up-regulated in tumor	0.06
PIWIL2	−2.51	Down-regulated in tumor	6.97E-5
PIWIL3	1.44	Up-regulated in tumor	0.12
PIWIL4	−1.95	Down-regulated in tumor	0.0044

**Table 4.** Of the four human homologs of PIWI gene, PIWIL1 and PIWIL3 were up-regulated but were not statistically significant. PIWIL2 and PIWIL4 genes were down-regulated and were statistically significant with p < 0.05.

**Table 5:** Univariate and multivariate results of PIWI genes

Parameter	Overall Survival				Recurrence Free Survival			
	Univariate analysis		Multivariate analysis		Univariate analysis		Multivariate analysis	
	HR (95% CI)	p-value	HR (95% CI)	p-value	HR (95% CI)	p-value	HR (95% CI)	p-value
**Risk score (for OS) PIWIL3 (for RFS)**	2.82 (1.49 – 5.33)	0.002	2.19 (1.14 – 4.22)	0.02	2.07 (1.17 – 3.64)	0.01	2.09 (1.18 – 3.71)	0.01
**Tumor stage**	0.62 (0.24 – 1.57)	0.31			0.56 (0.28 – 1.11)	0.09		
**Tumor grade**	2.31 (1.1 – 4.83)	0.03			1.75 (1.06 – 2.9)	0.03		
**Age at diagnosis**	1.04 (1.02 – 1.07)	0.001	1.04 (1.02 – 1.07)	0.001	1.01 (0.99 – 1.03)	0.22		
**TNBC status**	3.33 (1.77 – 6.26)	0.0002	2.35 (1.15 – 4.79)	0.02	1.72 (1.07 – 2.79)	0.03		

HR = Hazards ratio, CI = Confidence interval

**Table 5.** Univariate analysis was performed, considering PIWI genes as continuous variables. Two PIWI genes were significant for OS with p ≤ 0.15 and were used for constructing a risk score, PIWIL3 alone was significant for RFS with p ≤ 0.15. Risk score for OS and PIWIL3 for RFS were considered as categorical variables and were found to be significant in univariate and multivariate analyses using Cox proportional hazards regression model.

### piRNAs inhibit gene expression

Recent evidence suggests that piRNAs, in a mechanism similar to miRNAs, may regulate gene expression through base pair complementarity. However, very few studies have identified the corresponding gene targets for specific piRNAs [19, 20]. For this study, we only considered prognostically significant piRNAs (eight non–redundant piRNAs in total from OS and RFS) and focused on the inverse correlations between piRNA and its targets. Of the eight piRNAs, only six were differentially expressed (all were more than 1.5 FC) and were of immediate interest for target predictions. Since all six were up-regulated in tumors, relative to normal tissues, we extracted the 3′UTR sequences of all the down–regulated genes (n = 2,735) identified in our gene expression dataset. Using miRanda algorithm v3.3a and applying the cut–offs, we identified a total of 306 non–redundant gene targets for six piRNAs (Supplementary Tables S5A–S5F). We did not consider matched samples (between the piRNA data and the mRNA data) alone for target prediction, but instead utilized all the samples from our gene expression dataset since our previous study on miRNA-mRNA target identifications using the same mRNA dataset did not reveal profound differences between matched and unmatched datasets in the overall functional terms identified for the targets [28]. The identified gene targets were enriched for angiogenesis, transcription, cell signaling, cytoskeleton organization, membrane transport and organization (Supplementary Table S6).

## DISCUSSION

In this study, we have identified eight non-redundant piRNAs as novel prognostic markers for breast cancer. Four and six piRNAs were found to be associated with OS and RFS, respectively, among which two piRNAs were common for OS and RFS. We also successfully validated the prognostic significance of piRNAs associated with OS in an external dataset (TCGA). Gene targets for possible regulation by candidate piRNAs have also been identified. Although PIWI proteins have been studied by others as prognostic/diagnostic markers for other cancer types, their prognostic relevance in breast cancer has not been examined. In our study, we demonstrate the association of PIWI genes (as a proxy for PIWI proteins) with OS and RFS for breast cancer. Overall, this is the first study to comprehensively understand the significance of piRNAs and PIWI genes as prognostic markers for breast cancer using large and independent datasets with complete clinical annotation and a long follow–up period. In all, we have successfully captured the pathway of events and individual entities up-stream and down-stream of the piRNA biogenesis.

A new class of small non-coding RNAs called piRNAs was discovered in mouse testes in 2006 [2–5]. They were found to be involved in maintaining genome stability by regulating the expression of transposons in germ cells [6], and for a long time, their roles beyond germ cells remained uncertain. However, with increasing focus on these molecules, their presence in somatic cells has been observed and their functional roles are beginning to be uncovered. Using a sequencing platform to profile piRNAs, we observed the presence of 676 piRNAs in breast tissues, confirming their existence in somatic tissues. In contrast to their occurrence as clusters in germ cells, they were found to map to known transcripts in somatic cells. In breast tissues alone, we noted that around 85% (576 of the 676 total piRNAs profiled in our dataset) of the piRNAs mapped to exons and introns of known protein coding and non-coding transcripts (Figure 1C). Since piRNAs abundantly map to known genes, it remains to be determined if they are dependent on the host gene's promoter for their transcription or if they carry their own promoter.

The clinical relevance of piRNAs was first apparent when they were reported to be associated with clinicopathological factors such as lymph node status [23], and TNM stage [24]. Nonetheless, our understanding of their contribution as prognostic markers is rudimentary and warrants further exploration. We identified eight piRNAs as novel prognostic markers for breast cancer. To date, there has only been one study that has utilized sequencing data to interrogate piRNAs for breast cancer prognosis [26]. In the study by Martinez et al., piRNAs associated with OS were identified for eleven cancer types, including breast cancer. Our study is therefore the first to identify piRNAs associated with RFS as well as OS. We compared our eight prognostically significant piRNAs with their study findings and found that hsa_piR_009051 and hsa_piR_017061 were prognostically significant for renal clear cell carcinoma and colon adenocarcinoma, respectively. hsa_piR_021032 was significantly associated with renal clear cell carcinoma and lung squamous cell carcinoma prognoses. Significance of the remaining five piRNAs in cancer prognosis remains unknown till date.

An important observation from our study is that we may obtain a holistic picture of piRNAs associated with outcomes if we adopt a case–only approach. Case-control approach focuses on identifying prognostic markers which are differentially expressed [29, 30]. However, case-only approach interrogates the entire dataset in an unbiased manner [31–33] and may thus yield higher number of prognostic markers. We observed the same in our study, where, with the case–only method, we obtained four and six piRNAs for OS and RFS, respectively as opposed to three piRNAs each for OS and RFS. The piRNAs identified in the case–only approach included the ones identified from the case–control approach as well (Supplementary Tables S4A and S4B). Therefore, adopting a case–only approach may provide a more comprehensive understanding of the markers under investigation.

Another major finding of our study was the identification of genes coding for PIWI proteins as potential prognostic markers for breast cancer. Of the four human homologues of PIWI genes, two genes (PIWIL3 and PIWIL4) showed associations with OS, and PIWIL3 alone showed association with RFS (Table 5, Figure 5A and 5B). Reports on the clinical significance of PIWIL3 and PIWIL4 remain scarce [34–36], and in particular, this is the first study to identify the contribution of PIWIL3 and PIWIL4 genes to breast cancer prognosis. Further replication studies are warranted to better define their prognostic roles.

The functional importance of PIWI proteins and piRNAs is no longer restricted to the regulation of transposons or the maintenance and development of stem and germ cells. Based on previous studies that piRNAs inhibit gene expression, analogous to miRNAs [19, 20], we identified 306 gene targets (and their roles) for six piRNAs using our in-house gene expression dataset (Supplementary Tables S5 and S6). We did not restrict our analysis to gene ontology terms alone that identified terms related to cancer. We looked at the targets identified for every piRNA individually and found piRNA-mRNA pairs playing important roles in methylation, oxidative stress, and cell adhesion, among others (Supplementary Tables S5 and S6), the deregulation of which may contribute to an imbalance in cellular homeostasis. An interesting observation was that hsa_piR_021032 shared complementary sequence with PIWIL2. While PIWIL2 was down–regulated in our gene expression dataset, hsa_piR_021032 showed up-regulation in the tumor tissues, suggesting a possible repression of the PIWI gene by the piRNA. This proposed mechanism of PIWI regulation by piRNAs is novel and requires further validation.

Using a cohort with complete clinical annotation and long–term follow–up, we identified piRNAs and PIWI genes as novel prognostic markers for breast cancer. Identifying piRNA gene targets from breast tissue datasets is rare in the literature, and this study may open up research on the characterization of these piRNA–mRNA pairs. Deregulation of piRNAs and the involvement of the identified targets in key cellular mechanisms suggest that piRNAs may be important contributors to breast tumorigenesis. This is also the first time that we have observed a possible regulatory mechanism of PIWI genes by piRNAs, but it remains to be established if this regulation is through direct interaction or a complex network. Biomarker studies on piRNAs and PIWI genes and proteins are promising fields of research. Since piRNAs have exhibited stability in body fluids such as blood [37], serum and plasma [38], they may also serve as effective circulating biomarkers. With improving profiling platforms, availability of clinical samples with extensive clinical annotations will likely contribute to identification of additional piRNAs, furthering our understanding of their mechanistic and prognostic contributions to breast cancer and other diseases.

## MATERIALS AND METHODS

### Discovery cohort samples

Breast tumor tissues (stored as formalin fixed paraffin embedded blocks, FFPE) from 104 patients were obtained from Alberta Cancer Research Biobank (http://www.acrb.ca/). A detailed summary of the clinical characteristics of these samples, including information on tumor cellularity is given in our previous study [28]. Briefly, all the samples in our discovery cohort showed >70% cellularity in tumors, compared to ~60% of the samples from TCGA. Of the samples chosen for the study, 46 patients died and 61 patients underwent relapse. 11 breast tissues (stored as fresh frozen tissues, FF) were obtained from patients undergoing reduction mammoplasty and were considered as optimal controls for reasons elucidated elsewhere [28]. The number of samples used in both the groups were sufficient to conduct the study with 80% power, α = 0.05 and to identify piRNAs with a fold difference of 2 or more [http://bioinformatics.mdanderson.org/MicroarraySampleSize/, http://linus.nci.nih.gov/brb/samplesize/] [39, 40]. The study was approved by the local Institutional Research Ethics Committee (Health Research Ethics Board of Alberta–Cancer Committee) and written informed consent was obtained from all the study subjects.

### Genome-wide profiling of piRNAs

Data generated for the study is deposited in Gene Expression Omnibus (GEO accession ID GSE68085). Small RNA libraries for next generation sequencing experiment (NGS) were generated for all the samples individually using their total RNA. We have already described in detail the RNA isolation and sequencing protocols that we followed [28]. Briefly, 36 cycles single end protocol was applied in Illumina Genome analyzer IIx platform, followed by base calling and demultiplexing using CASAVA 1.8.2 and adapter trimming using cutadapt software [41]. Of these 36 nucleotides, 7 belonged to the index sequence, leaving behind 29 nt. We had initially focused on reads with a length ranging from 17 to 27 nt. Nevertheless, the piRNAs annotated in this dataset (n = 676) included even the longer piRNAs (29-32 nt). Reads trimmed of adapters were aligned to hg 19 genomic assembly (downloaded from Illumina iGenome repository) using Bowtie [42]. In the quality control process, one sample was deemed unusable and was removed from further processing. Memory efficient .bam files generated from .sam files served as input files for further analysis using Partek Genomics Suite 6.6 (PGS, Partek® Genomics Suite software, version 6.6 beta, Copyright © 2009 Partek Inc., St. Louis, MO, USA). piRNAs were annotated using piRNA bank (http://pirnabank.ibab.ac.in/index.shtml) [27]. For all the analyses (explained below), raw data was normalized using reads per kilobase per million (RPKM) method [43] and potential sample outliers were removed based on principal component analysis clustering.

### Identification of piRNAs as prognostic markers using two statistical methods

Two statistical approaches were adopted for our study: Case-control (CC) and Case-only (CO). The difference between the two statistical models lies in the process of selecting piRNAs for survival analysis. The CC paradigm is one of the most commonly used methods that concentrates on the prognostic potential of differentially expressed (DE) piRNAs exhibiting > 2 fold change (FC) and a false discovery rate (FDR) cut off of 0.05 (one-way ANOVA). In contrast, CO method is unbiased, i.e., it includes all of the piRNAs profiled in the tumor samples and is not influenced by expression differences between normal and tumor samples, thus eliminates the bias introduced by the definition of a normal sample.

In both approaches, we applied a stringent threshold to select only those piRNAs with ≥ 10 read counts in 90% of the samples (tumor and normal inclusive in CC and only tumor samples in CO) for downstream analysis; the data was also adjusted for potential batch effects. piRNA datasets from both the methods (DE piRNAs from CC and all the filtered piRNAs from CO) were subjected to univariate Cox proportional hazards regression model for OS and RFS using SAS (SAS institute Inc., Cary, NC) version 9.3, followed by permutation test using R statistical program (package - ‘glmperm’). Further, risk score was constructed for every sample using piRNAs significant with a permuted p-value ≤ 0.1. Receiver operating characteristics curve (ROC) was employed for estimating optimal cut-off points for both the outcomes (two for CC and two for CO) to stratify patients into low and high-risk groups. Subsequently, multivariate Cox regression model was performed and where appropriate, age at diagnosis (continuous variable), tumor stage (I, II vs. III and IV), grade (high vs. low) and Triple Negative Breast Cancer status (Luminal vs. TNBC, since our sample composition is from these two subtypes) were considered as potential confounders. Hazards ratio (HR) and confidence interval (CI) are reported as univariate and multivariate test results. Probability of survival over a given length of time was computed using Kaplan-Meier method and survival differences between the two risk groups were estimated using log-rank test. For all the tests, p< 0.05 was considered to be statistically significant. The overall workflow of the study was described in detail in our previous study [28].

### External validation of piRNA signatures of prognostic significance

Following stringent filtering criteria (summarized elsewhere [28]), 84 samples were accessed from The Cancer Genome Atlas Project (TCGA), which is an international consortium that generates genome datasets from diverse geographical locations. Alignment (.bam) files of 84 samples were analyzed using PGS and similar to discovery cohort data, this data was corrected for batch ID, plate ID and tissue source site. Analysis for OS was conducted with 27 events (deaths). However, the same could not be done for RFS since the information on breast cancer recurrence was not sufficient in the TCGA dataset. RPKM normalized counts of the piRNAs identified for OS in the discovery cohort were extracted and utilized for constructing risk score. Univariate and multivariate Cox regression analyses were performed with available clinical information, as explained for the discovery cohort. The discovery and validation cohorts differed in several aspects, including tumor cellularity and have been reported in our previous study [28]. We observed that despite these differences, the identified signatures showed similar trends in their direction of effects in both the datasets.

### PIWI genes as prognostic markers for breast cancer

The in-house gene (mRNA) expression dataset generated using Agilent microarray platform for ten normal breast tissues (obtained from reduction mammoplasty) and 141 breast tumor tissues was accessed from gene expression omnibus (GSE22820) [44]. The data was quantile normalized and log2 transformed using PGS. Differential expression analysis was performed using one-way ANOVA to observe the expression patterns of the four human homologues of PIWI genes (PIWIL1 – PIWIL4). Survival analysis was performed for OS and RFS since we had 42 deaths and 77 recurrence events in our dataset. Treating the four genes as continuous variables, univariate Cox regression analysis was carried out; PIWI genes with p ≤ 0.15 were used for constructing a risk score and ROC estimated the optimal cut-off point for patient stratification into low and high-risk groups. Risk score was then treated as dichotomous variable; univariate and multivariate analysis was performed, considering tumor stage, grade, age at diagnosis and TNBC status as potential confounders.

### Identification of gene targets for significant piRNAs and their functional roles

Of the eight prognostically significant piRNAs, six were DE and were of immediate interest for the gene target prediction. Recent evidence has suggested (i) interaction between piRNAs and mRNAs through base-pair complementarity and (ii) a possible inverse correlation between piRNA expression and its corresponding mRNA targets [19, 20]. Since all the six piRNAs (selected for target prediction) were up-regulated, only down-regulated genes (mRNAs), with FC > 2.0 and FDR 0.05 (as determined by one-way ANOVA) were extracted from the in-house gene expression dataset. The breast tissues (tumor tissue and normal reduction mammoplasty specimens) used in both our NGS and mRNA expression experiments are from the same clinics in Alberta. We have demonstrated earlier utility of these datasets to interrogate correlations between miRNA and mRNA expressions [28] and focused initially on the putative binding of piRNAs to 3′ UTRs of coding genes, even though other possible mechanisms of action have been suggested, viz., coding exons and 5′UTRs [20]. Fasta sequences of the 3′UTR of all the down-regulated genes were obtained from Ensembl database (GRCh37) and fasta sequences of the six piRNAs were obtained from piRNA Bank (hg 19). As such, there are no target prediction databases available for piRNAs. However, predictions based on the list of input genes (in our study, down-regulated genes in breast cancer tissues) were obtained using miRanda v 3.3a algorithm [45], with alignment score ≥ 170 and energy threshold ≤ −20 kcal/mol [20]. Potential functional insights of the targets (with a focus on biological processes) identified were obtained using DAVID bioinformatics tool (http://david.abcc.ncifcrf.gov/) [46] and we report gene ontology (GO) terms related to cancer with p < 0.05 in the current study.

## SUPPLEMENTARY FIGURE AND TABLES








