# Supplementary material for: Piwi-interacting RNAs and PIWI genes as novel prognostic markers for breast cancer
Source: Oncotarget. 2016 May 10;7(25):37944–56. doi: 10.18632/oncotarget.9272 (PMC5122362; doi:10.18632/oncotarget.9272)
Supplement: Supplementary file 1 [file oncotarget-07-37944-s001.pdf]

Piwi-interacting RNAs and PIWI genes as novel prognostic markers for breast cancer

SUPPLEMENTARY FIGURE AND TABLES

Supplementary Figure S1: Detection and correction of batch effects

A. Before batch correction **B. After batch correction**

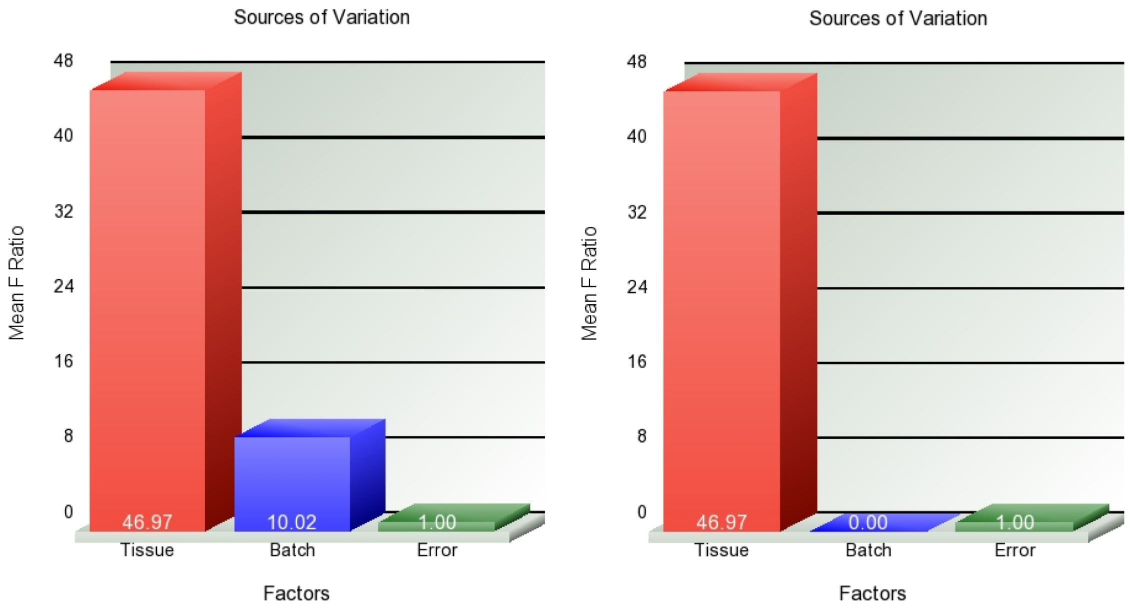

Supplementary Figure S1: Detection and correction of batch effects. Samples were sequenced in four batches and we detected batch effects (technical variation) in the RPKM normalized data (**A**), with mean F ratio of batch (blue bar) more than the error bar. ANOVA model was employed to correct for batch effects and in figure **B**, where mean F ratio of batch is less than 1, indicates that the data has been corrected for batch effects. Red bar indicates the variation arising from tissue source (normal and tumor) and since this is a biological variation, this was not corrected for.

Supplementary Table S1: Distribution of piRNAs in human genome

See Supplementary File 1

Supplementary Table S2: List of differentially expressed piRNAs

piRNA ID	Fold Change	Direction of fold change	p-value	FDR
hsa_piR_008114	-8.54402	Down-regulated in tumor	2.51E-31	6.29E-30
hsa_piR_019676	-8.01064	Down-regulated in tumor	1.10E-28	9.13E-28
hsa_piR_000552	-6.55583	Down-regulated in tumor	1.68E-30	2.10E-29
hsa_piR_020548	-4.89823	Down-regulated in tumor	1.22E-27	7.62E-27
hsa_piR_008113	-3.70784	Down-regulated in tumor	2.88E-20	1.44E-19
hsa_piR_016735	-2.87514	Down-regulated in tumor	2.52E-14	9.01E-14
hsa_piR_020450	-2.74341	Down-regulated in tumor	2.59E-11	7.20E-11
hsa_piR_017033	-2.16751	Down-regulated in tumor	0.00085	0.001
hsa_piR_020365	2.2346	Up-regulated in tumor	0.026	0.026
hsa_piR_019675	2.32945	Up-regulated in tumor	7.19E-09	1.80E-08
hsa_piR_019914	2.41544	Up-regulated in tumor	0.007	0.007
hsa_piR_015249	2.42369	Up-regulated in tumor	1.21E-13	3.77E-13
hsa_piR_009294	2.95468	Up-regulated in tumor	3.78E-05	5.25E-05
hsa_piR_021032	3.60869	Up-regulated in tumor	1.10E-05	1.72E-05
hsa_piR_009051	4.38365	Up-regulated in tumor	1.32E-06	2.53E-06
hsa_piR_000753	5.84609	Up-regulated in tumor	0.013	0.014
hsa_piR_008112	6.93714	Up-regulated in tumor	1.50E-08	3.41E-08
hsa_piR_020814	7.14901	Up-regulated in tumor	2.29E-06	4.09E-06
hsa_piR_001318	8.70609	Up-regulated in tumor	3.55E-06	5.91E-06
hsa_piR_006426	8.94062	Up-regulated in tumor	0.002	0.003
hsa_piR_017184	9.17035	Up-regulated in tumor	3.76E-07	7.84E-07
hsa_piR_020829	9.57916	Up-regulated in tumor	1.63E-05	2.39E-05
hsa_piR_019912	16.6365	Up-regulated in tumor	1.56E-15	6.50E-15
hsa_piR_018780	18.4532	Up-regulated in tumor	8.63E-05	0.0001
hsa_piR_018849	27.2571	Up-regulated in tumor	0.023	0.024

FDR = False discovery rate.

Supplementary Table S3: Raw and normalized counts of all the piRNAs profiled

See Supplementary File 2

Supplementary Table S4: piRNAs significant for OS and RFS in univariate Cox analysis

A. Overall Survival

piRNA ID	Univariate Cox p-value	Permuted p-value
hsa_piR_009051	0.01	0.01
hsa_piR_021032	0.01	0.03
hsa_piR_015249	0.06	0.07
hsa_piR_020541	0.07	0.09

B. Recurrence Free Survival

piRNA ID	Univariate Cox p-value	Permuted p-value
hsa_piR_017061	0.02	0.02
hsa_piR_009051	0.03	0.05
hsa_piR_021032	0.03	0.06
hsa_piR_004153	0.08	0.06
hsa_piR_017716	0.09	0.08
hsa_piR_019914	0.09	0.09

Supplementary Table S4: Overall, four piRNAs and six piRNAs were significant for OS (A) and RFS (B), respectively from the CO approach. However, the CO approach identified RNAs also included all of the piRNAs significant in the CC approach (three for OS and three for RFS) and are indicated in red color.

Supplementary Table S5A: List of gene targets for hsa_piR_009051

Supplementary Table S5B: List of gene targets for hsa_piR_021032

Supplementary Table S5C: List of gene targets for hsa_piR_015249

Supplementary Table S5D: List of gene targets for hsa_piR_004153

Supplementary Table S5E: List of gene targets for hsa_piR_017716

Supplementary Table S5F: List of gene targets for hsa_piR_019914

See Supplementary File 3

Supplementary Table S6: Identification of piRNA gene targets and their functional roles

piRNA ID	# of gene targets	# of GO clusters	Targets	GO term
hsa_piR_009051	10	1	SSBP2, FOXO4, NR5A2, ZNF177, ZNF765	Regulation of transcription
hsa_piR_021032	180	27	KCNMA1, CAV2, NRP1, SCN2B, GLRA3, AKAP9, NRXN1, ATP1A2, ESR2, PARK2, KCNMB1, SEMA5A, LEP, PDE7B, NPTX1, KIF1B, KCNN1, SLC22A3	Cell-cell signaling
			SEMA5A, NRP1, PLXDC1, LEPR, CCBE1, ROBO4, TNFSF12	Angiogenesis
			KCNMA1, TRPM3, TRPM6, CUBN, SLC16A12, ATP1A2, SLC26A4, SLC2A4, SLC22A3, SLC25A37, KCNH8, SV2B, NALCN, SLC25A26	Transmembrane transport
			TXNIP, KCNMA1, CAV2, GSTM3, LEPR	Response to estrogen stimulus
			TRIOBP, SHROOM4, MRAS, NEDD9, FGD5, ARHGAP26, FGD4	Actin cytoskeleton organization
			LEP, LEPR, GAB1, PDCD4, FGD4	Regulation of MAPKKK cascade
			GAB1, PDCD4, FGD4	Regulation of JUN kinase activity
hsa_piR_015249	1	0	FOXP2	Transcription
hsa_piR_004153	42	6	ALPL, CALCR, CAV1	Response to glucocorticoid stimulus
hsa_piR_017716	72	7	ALPL, PPARA, GNG2, FOXO4, ACVR1C, SLC34A2	Response to hormone stimulus
			LAMA4, EPAS1, TNFSF12, ANGPTL4	Blood vessel development
			KCNK17, SLC23A2, P2RX3, KCNMB1, SLC34A2, ATP13A4, GRID1	Ion transport
hsa_piR_019914	45	7	EREG, LEPR, PLCD3, CXCL12	Angiogenesis
			EREG, LEPR, IGF1, GHR	Positive regulation of signal transduction
			LY75, ARRB1, EHD2, GHR	Membrane organization

GO = Gene Ontology; GO clusters represent biological processes; GO term includes cancer related terms with $p < 0.05$.
