# Supplementary material for: Piwi-interacting RNAs and PIWI genes as novel prognostic markers for breast cancer
Source: Oncotarget. 2016 May 10;7(25):37944–56. doi: 10.18632/oncotarget.9272 (PMC5122362; doi:10.18632/oncotarget.9272)
Supplement: Supplementary file 4 [file oncotarget-07-37944-s004.docx]

**Supplementary Table S5A. List of gene targets for hsa_piR_009051**

| **Gene targets for hsa_piR_009051** | **Alignment score** | **Energy score** | **Gene name** |
| --- | --- | --- | --- |
| NR5A2 | 176 | -20.08 | nuclear receptor subfamily 5, group A, member 2 |
| ZNF177 | 175 | -20.46 | zinc finger protein 177 |
| SSBP2 | 179 | -23.62 | single-stranded DNA binding protein 2 |
| KCNS1 | 170 | -23.96 | Potassium Voltage-Gated Channel, Delayed-Rectifier, Subfamily S, Member 1 |
| ZNF765 | 171 | -24.68 | zinc finger protein 765 |
| TMCC3 | 170 | -25.63 | transmembrane and coiled-coil domain family 3 |
| FOXO4 | 177 | -26.89 | forkhead box O4 |
| TRIM2 | 173 | -28.11 | tripartite motif containing 2 |
| SCARA3 | 178 | -28.79 | scavenger receptor class A, member 3 |
| KCNA1 | 171 | -32.74 | potassium voltage-gated channel, shaker-related subfamily, member 1 (episodic ataxia with myokymia) |

**Supplementary Table S5B. List of gene targets for hsa_piR_021032**

| **Gene targets for hsa_piR_021032** | **Alignment score** | **Energy score** | **Gene name** |
| --- | --- | --- | --- |
| COL4A3 | 170 | -21.43 | collagen, type IV, alpha 3 (Goodpasture antigen) |
| SOBP | 170 | -21.79 | sine oculis binding protein homolog (Drosophila) |
| ZBTB33 | 170 | -21.86 | zinc finger and BTB domain containing 33 |
| PKN2 | 170 | -22.46 | protein kinase N2 |
| ADHFE1 | 170 | -22.62 | alcohol dehydrogenase, iron containing, 1 |
| IRX4 | 170 | -23.67 | iroquois homeobox 4 |
| GTF2IRD2 | 170 | -24.04 | GTF2I repeat domain containing 2 |
| RNF213 | 170 | -25.02 | ring finger protein 213 |
| EPDR1 | 170 | -31.49 | ependymin related 1 |
| RNF180 | 171 | -20.16 | ring finger protein 180 |
| ZNF333 | 171 | -21.6 | zinc finger protein 333 |
| ADAM33 | 171 | -21.73 | ADAM metallopeptidase domain 33 |
| FBXO9 | 171 | -21.73 | F-box protein 9 |
| LRRC28 | 171 | -21.73 | leucine rich repeat containing 28 |
| SEMA5A | 171 | -21.73 | sema domain, seven thrombospondin repeats (type 1 and type 1-like), transmembrane domain (TM) and short cytoplasmic domain, (semaphorin) 5A |
| ZNF506 | 171 | -21.73 | zinc finger protein 506 |
| CIRBP | 171 | -22.38 | cold inducible RNA binding protein |
| SMAD9 | 171 | -22.51 | SMAD family member 9 |
| FRMD4A | 171 | -23.34 | FERM domain containing 4A |
| SYNPO2 | 171 | -23.55 | synaptopodin 2 |
| ARHGAP26 | 171 | -23.56 | Rho GTPase activating protein 26 |
| CTDSPL | 171 | -23.56 | CTD (carboxy-terminal domain, RNA polymerase II, polypeptide A) small phosphatase-like |
| SLC25A26 | 171 | -23.56 | solute carrier family 25 (S-adenosylmethionine carrier), member 26 |
| VLDLR | 171 | -23.77 | very low density lipoprotein receptor |
| MAPKBP1 | 171 | -25.57 | mitogen-activated protein kinase binding protein 1 |
| PTCHD1 | 171 | -25.57 | patched domain containing 1 |
| TPT1 | 171 | -26.34 | tumor protein, translationally-controlled 1 |
| LDB3 | 171 | -26.82 | LIM domain binding 3 |
| MYOZ3 | 171 | -27.33 | myozenin 3 |
| CRTAP | 171 | -27.67 | cartilage associated protein |
| GNAI1 | 171 | -29.84 | guanine nucleotide binding protein (G protein), alpha inhibiting activity polypeptide 1 |
| FGD5 | 172 | -21.2 | FYVE, RhoGEF and PH domain containing 5 |
| KIRREL | 172 | -22.49 | kin of IRRE like (Drosophila) |
| TGFB1I1 | 172 | -22.63 | transforming growth factor beta 1 induced transcript 1 |
| LTBP2 | 172 | -24.17 | latent transforming growth factor beta binding protein 2 |
| SV2B | 172 | -24.82 | synaptic vesicle glycoprotein 2B |
| ZNF488 | 172 | -25.19 | zinc finger protein 488 |
| KIF1B | 172 | -25.49 | kinesin family member 1B |
| TLE4 | 172 | -26.4 | transducin-like enhancer of split 4 |
| PER2 | 172 | -26.64 | period circadian clock 2 |
| KLHL3 | 172 | -28.05 | kelch-like family member 3 |
| TMEM64 | 173 | -23.15 | transmembrane protein 64 |
| VSX1 | 173 | -24.08 | visual system homeobox 1 |
| TRIOBP | 173 | -24.09 | TRIO and F-actin binding protein |
| ST8SIA3 | 173 | -28.16 | ST8 alpha-N-acetyl-neuraminide alpha-2,8-sialyltransferase 3 |
| PPARA | 174 | -23.49 | peroxisome proliferator-activated receptor alpha |
| TRPM3 | 174 | -23.78 | transient receptor potential cation channel, subfamily M, member 3 |
| DNAJC18 | 174 | -24.29 | DnaJ (Hsp40) homolog, subfamily C, member 18 |
| PPP1R12B | 174 | -24.43 | protein phosphatase 1, regulatory subunit 12B |
| MYBPC1 | 174 | -25.16 | myosin binding protein C, slow type |
| NPTX1 | 174 | -25.18 | neuronal pentraxin I |
| FGFR1 | 174 | -25.55 | fibroblast growth factor receptor 1 |
| SLC16A12 | 174 | -26.16 | solute carrier family 16, member 12 |
| NBEA | 174 | -28.54 | neurobeachin |
| ALX4 | 174 | -29.2 | ALX homeobox 4 |
| TNRC6B | 175 | -22.28 | trinucleotide repeat containing 6B |
| FOSB | 175 | -25.06 | FBJ murine osteosarcoma viral oncogene homolog B |
| LEPR | 175 | -25.14 | leptin receptor |
| BVES | 175 | -25.47 | **BVES** antisense RNA 1 |
| PIWIL2 | 175 | -25.7 | piwi-like RNA-mediated gene silencing 2 |
| SHROOM4 | 175 | -26.35 | shroom family member 4 |
| MSRB3 | 175 | -26.89 | methionine sulfoxide reductase B3 |
| CLDN19 | 175 | -27.23 | claudin 19 |
| ICA1L | 175 | -27.23 | islet cell autoantigen 1,69kDa-like |
| KLK10 | 175 | -27.46 | kallikrein-related peptidase 10 |
| METTL7A | 175 | -27.46 | methyltransferase like 7A |
| PDE7B | 175 | -27.46 | phosphodiesterase 7B |
| MRAS | 175 | -27.62 | muscle RAS oncogene homolog |
| ART4 | 175 | -27.97 | ADP-ribosyltransferase 4 (Dombrock blood group) |
| CAV2 | 175 | -27.97 | caveolin 2 |
| MAN2A2 | 175 | -28.33 | mannosidase, alpha, class 2A, member 2 |
| TXNIP | 175 | -31.5 | thioredoxin interacting protein |
| CFL2 | 176 | -24.27 | cofilin 2 (muscle) |
| NALCN | 177 | -27.04 | sodium leak channel, non-selective |
| FGD4 | 178 | -26.18 | FYVE, RhoGEF and PH domain containing 4 |
| RPL13 | 178 | -27.15 | ribosomal protein L13 |
| HLF | 178 | -27.64 | hepatic leukemia factor |
| KCNMA1 | 179 | -21.93 | potassium large conductance calcium-activated channel, subfamily M, alpha member 1 |
| POLR1E | 179 | -25.26 | polymerase (RNA) I polypeptide E, 53kDa |
| AKAP9 | 179 | -25.79 | A kinase (PRKA) anchor protein 9 |
| FANCC | 179 | -25.79 | Fanconi anemia, complementation group C |
| SETD5 | 179 | -26.06 | SET domain containing 5 |
| NEDD9 | 179 | -26.79 | neural precursor cell expressed, developmentally down-regulated 9 |
| ATP1A2 | 179 | -27.15 | ATPase, Na+/K+ transporting, alpha 2 polypeptide |
| AP3B2 | 179 | -27.15 | adaptor-related protein complex 3, beta 2 subunit |
| CEP68 | 179 | -27.15 | centrosomal protein 68kDa |
| CCBE1 | 179 | -27.15 | collagen and calcium binding EGF domains 1 |
| DIXDC1 | 179 | -27.15 | DIX domain containing 1 |
| CPM | 179 | -27.15 | carboxypeptidase M |
| FGFR2 | 179 | -27.15 | fibroblast growth factor receptor 2 |
| KRT15 | 179 | -27.15 | keratin 15 |
| MICAL3 | 179 | -27.15 | microtubule associated monooxygenase, calponin and LIM domain containing 3 |
| PDCD4 | 179 | -27.15 | programmed cell death 4 (neoplastic transformation inhibitor) |
| NRIP2 | 179 | -27.15 | nuclear receptor interacting protein 2 |
| PCGF5 | 179 | -27.15 | polycomb group ring finger 5 |
| PARK2 | 179 | -27.15 | parkin RBR E3 ubiquitin protein ligase |
| NRP1 | 179 | -27.15 | neuropilin 1 |
| SLC26A4 | 179 | -27.15 | solute carrier family 26 (anion exchanger), member 4 |
| SLC25A37 | 179 | -27.15 | solute carrier family 25 (mitochondrial iron transporter), member 37 |
| STAC2 | 179 | -27.15 | SH3 and cysteine rich domain 2 |
| ST13 | 179 | -27.15 | suppression of tumorigenicity 13 (colon carcinoma) (Hsp70 interacting protein) |
| ZNF667 | 179 | -27.15 | zinc finger protein 667 |
| UACA | 179 | -27.15 | uveal autoantigen with coiled-coil domains and ankyrin repeats |
| ZNF483 | 179 | -27.15 | zinc finger protein 483 |
| PKD2 | 179 | -28.77 | polycystic kidney disease 2 (autosomal dominant) |
| GAB1 | 179 | -28.9 | GRB2-associated binding protein 1 |
| CLPX | 179 | -28.98 | caseinolytic mitochondrial matrix peptidase chaperone subunit |
| CHNRB1 | 179 | -28.98 |  |
| CD160 | 179 | -28.98 | **CD160** molecule |
| CCDC69 | 179 | -28.98 | coiled-coil domain containing 69 |
| ESR2 | 179 | -28.98 | estrogen receptor 2 (ER beta) |
| HB3ST1 | 179 | -28.98 |  |
| GSTM3 | 179 | -28.98 | glutathione S-transferase mu 3 (brain) |
| GLRA3 | 179 | -28.98 | glycine receptor, alpha 3 |
| GNAL | 179 | -28.98 | guanine nucleotide binding protein (G protein), alpha activating activity polypeptide, olfactory type |
| MCAM | 179 | -28.98 | melanoma cell adhesion molecule |
| LEP | 179 | -28.98 | leptin |
| LYRM7 | 179 | -28.98 | LYR motif containing 7 |
| OTUD6A | 179 | -28.98 | OTU deubiquitinase 6A |
| NMT2 | 179 | -28.98 | N-myristoyltransferase 2 |
| RNF157 | 179 | -28.98 | ring finger protein 157 |
| SLC14A2 | 179 | -28.98 | solute carrier family 14 (urea transporter), member 2 |
| SCN2B | 179 | -28.98 | sodium channel, voltage-gated, type II, beta subunit |
| ROBO4 | 179 | -28.98 | roundabout, axon guidance receptor, homolog 4 (Drosophila) |
| SOX7 | 179 | -28.98 | SRY (sex determining region Y)-box 7 |
| SMYD4 | 179 | -28.98 | SET and MYND domain containing 4 |
| SEMA3E | 179 | -28.98 | sema domain, immunoglobulin domain (Ig), short basic domain, secreted, (semaphorin) 3E |
| SAMD5 | 179 | -28.98 | sterile alpha motif domain containing 5 |
| RPS9 | 179 | -28.98 | ribosomal protein S9 |
| SH3TC2 | 179 | -28.98 | SH3 domain and tetratricopeptide repeats 2 |
| SVEP1 | 179 | -28.98 | sushi, von Willebrand factor type A, EGF and pentraxin domain containing 1 |
| TBRG1 | 179 | -28.98 | transforming growth factor beta regulator 1 |
| TCF7 | 179 | -28.98 | transcription factor 7 (T-cell specific, HMG-box) |
| TRPM6 | 179 | -28.98 | transient receptor potential cation channel, subfamily M, member 6 |
| TMEM130 | 179 | -28.98 | transmembrane protein 130 |
| ZNF154 | 179 | -28.98 | zinc finger protein 154 |
| SLC2A4 | 179 | -29.07 | solute carrier family 2 (facilitated glucose transporter), member 4 |
| LIAS | 179 | -29.08 | lipoic acid synthetase |
| RASSF6 | 179 | -29.08 | Ras association (RalGDS/AF-6) domain family member 6 |
| FILIP1 | 179 | -29.57 | filamin A interacting protein 1 |
| ANXA11 | 179 | -30.15 | annexin A11 |
| MON1B | 179 | -33.09 | MON1 secretory trafficking family member B |
| ZNF366 | 179 | -33.09 | zinc finger protein 366 |
| ZNF135 | 180 | -27.12 | zinc finger protein 135 |
| USHBP1 | 180 | -27.82 | Usher syndrome 1C binding protein 1 |
| BICC1 | 182 | -24.7 | BicC family RNA binding protein 1 |
| ACAT1 | 183 | -25.7 | acetyl-CoA acetyltransferase 1 |
| PCDH11Y | 183 | -26.05 | protocadherin 11 Y-linked |
| PAK3 | 183 | -26.05 | p21 protein (Cdc42/Rac)-activated kinase 3 |
| FUT6 | 183 | -27.89 | fucosyltransferase 6 (alpha (1,3) fucosyltransferase) |
| IDS | 183 | -27.89 | iduronate 2-sulfatase |
| KCNN1 | 183 | -27.89 | potassium intermediate/small conductance calcium-activated channel, subfamily N, member 1 |
| LRRC2 | 183 | -27.89 | leucine rich repeat containing 2 |
| LYRM4 | 183 | -27.89 | LYR motif containing 4 |
| SLC14A1 | 183 | -27.89 | solute carrier family 14 (urea transporter), member 1 (Kidd blood group) |
| ZHX3 | 183 | -27.89 | zinc fingers and homeoboxes 3 |
| ZNF561 | 183 | -27.89 | zinc finger protein 561 |
| XPNPEP3 | 183 | -28.12 | X-prolyl aminopeptidase (aminopeptidase P) 3, putative |
| CA5B | 183 | -28.89 | carbonic anhydrase VB, mitochondrial |
| KCNMB1 | 183 | -29.49 | potassium large conductance calcium-activated channel, subfamily M, beta member 1 |
| CHDH | 183 | -31.6 | choline dehydrogenase |
| SLC22A3 | 184 | -25.9 | solute carrier family 22 (organic cation transporter), member 3 |
| GGT6 | 184 | -26.33 | gamma-glutamyltransferase 6 |
| PLXDC1 | 184 | -31.84 | plexin domain containing 1 |
| CUBN | 184 | -32.53 | cubilin (intrinsic factor-cobalamin receptor) |
| FBXL2 | 185 | -26.74 | F-box and leucine-rich repeat protein 2 |
| KCNH8 | 185 | -28.44 | potassium voltage-gated channel, subfamily H (eag-related), member 8 |
| BHMT2 | 185 | -30.57 | betaine--homocysteine S-methyltransferase 2 |
| FER | 185 | -36.78 | **fer** (fps/fes related) tyrosine kinase |
| NRXN1 | 186 | -25.31 | neurexin 1 |
| NFAT5 | 187 | -29.16 | nuclear factor of activated T-cells 5, tonicity-responsive |
| PARD6G | 187 | -32.32 | par-6 family cell polarity regulator gamma |
| DENND2A | 189 | -30.46 | DENN/MADD domain containing 2A |
| TCEAL7 | 189 | -31.22 | transcription elongation factor A (SII)-like 7 |
| NOSTRIN | 190 | -31.24 | nitric oxide synthase trafficking |
| SHF | 191 | -30.12 | Src homology 2 domain containing F |
| EPB42 | 191 | -32.31 | erythrocyte membrane protein band 4.2 |
| ZNF589 | 191 | -32.31 | zinc finger protein 589 |
| ITIH5 | 192 | -31.93 | inter-alpha-trypsin inhibitor heavy chain family, member 5 |
| TNFSF12 | 197 | -39.56 | tumor necrosis factor (ligand) superfamily, member 12 |

**Supplementary Table S5C. List of gene targets for hsa_piR_015249**

| **Gene target for hsa_piR_015249** | **Alignment score** | **Energy score** | **Gene name** |
| --- | --- | --- | --- |
| FOXP2 | 174 | -21.46 | forkhead box P2 |

**Supplementary Table S5D. List of gene targets for hsa_piR_004153**

| **Gene targets for hsa_piR_004153** | **Alignment score** | **Energy score** | **Gene name** |
| --- | --- | --- | --- |
| IL22RA1 | 170 | -28.7 | Interleukin 22 Receptor, Alpha 1 |
| UST | 170 | -30.51 | uronyl-2-sulfotransferase |
| FOXP2 | 170 | -31.14 | forkhead box P2 |
| RPS9 | 170 | -31.78 | ribosomal protein S9 |
| LILRB5 | 170 | -39.16 | leukocyte immunoglobulin-like receptor, subfamily B (with TM and ITIM domains), member 5 |
| AFP | 171 | -26.72 | alpha-fetoprotein |
| MYOZ3 | 171 | -27.7 | myozenin 3 |
| ARHGAP19 | 171 | -31.16 | Rho GTPase activating protein 19 |
| APOL4 | 171 | -31.74 | apolipoprotein L, 4 |
| FRMD1 | 171 | -36.5 | FERM domain containing 1 |
| AGPAT2 | 171 | -39.15 | 1-acylglycerol-3-phosphate O-acyltransferase 2 |
| MLXIPL | 171 | -40.44 | MLX interacting protein-like |
| PLEKHA4 | 171 | -45.37 | pleckstrin homology domain containing, family A (phosphoinositide binding specific) member 4 |
| GRID1 | 172 | -29.15 | glutamate receptor, ionotropic, delta 1 |
| SCRT1 | 172 | -31.47 | scratch family zinc finger 1 |
| GRPR3 | 172 | -31.73 | gastrin-releasing peptide receptor |
| KCNMB1 | 172 | -35.46 | potassium large conductance calcium-activated channel, subfamily M, beta member 1 |
| AGBL5 | 172 | -36.39 | ATP/GTP binding protein-like 5 |
| DAB2IP | 172 | -41.26 | DAB2 interacting protein |
| CCDC38 | 173 | -25.29 | coiled-coil domain containing 38 |
| CTNND1 | 173 | -32.75 | catenin (cadherin-associated protein), delta 1 |
| CPN2 | 173 | -33.79 | carboxypeptidase N, polypeptide 2 |
| ZNF395 | 173 | -34.07 | zinc finger protein 395 |
| TMCC3 | 173 | -40.19 | transmembrane and coiled-coil domain family 3 |
| ITSN1 | 174 | -33.86 | intersectin 1 (SH3 domain protein) |
| CRHR1 | 174 | -33.99 | corticotropin releasing hormone receptor 1 |
| GLB1L3 | 174 | -36.2 | galactosidase, beta 1-like 3 |
| MGLL | 174 | -36.67 | monoglyceride lipase |
| CPEB1 | 174 | -38.76 | cytoplasmic polyadenylation element binding protein 1 |
| LAMA4 | 174 | -45.36 | laminin, alpha 4 |
| CALCR | 175 | -34.27 | calcitonin receptor |
| CYGB | 175 | -34.59 | cytoglobin |
| ST8SIA3 | 175 | -36.46 | ST8 alpha-N-acetyl-neuraminide alpha-2,8-sialyltransferase 3 |
| CLEC4F | 175 | -43.15 | C-type lectin domain family 4, member F |
| PIGR | 176 | -38.81 | polymeric immunoglobulin receptor |
| OLFML2A | 178 | -37.25 | olfactomedin-like 2A |
| LUZP1 | 178 | -39.93 | leucine zipper protein 1 |
| SHANK3 | 178 | -45.21 | SH3 and multiple ankyrin repeat domains 3 |
| ADAM11 | 181 | -42.41 | ADAM metallopeptidase domain 11 |
| CAV1 | 185 | -38.06 | caveolin 1, caveolae protein, 22kDa |
| H6PD | 188 | -40.62 | hexose-6-phosphate dehydrogenase (glucose 1-dehydrogenase) |
| ALPL | 193 | -36.67 | alkaline phosphatase, liver/bone/kidney |

**Supplementary Table S5E. List of gene targets for hsa_piR_017716**

| **Gene targets for hsa_piR_017716** | **Alignment score** | **Energy score** | **Gene name** |
| --- | --- | --- | --- |
| CLIP3 | 170 | -27.29 | CAP-GLY domain containing linker protein 3 |
| ERICH1 | 170 | -27.51 | glutamate-rich 1 |
| ACSM | 170 | -28.04 | acyl-CoA synthetase medium-chain family member 2B |
| TIFA | 170 | -30.23 | TRAF-interacting protein with forkhead-associated domain |
| SLC34A2 | 170 | -31.76 | solute carrier family 34 (type II sodium/phosphate contransporter), member 2 |
| PIK3IP1 | 170 | -32.25 | phosphoinositide-3-kinase interacting protein 1 |
| EPHA2 | 170 | -32.49 | EPH receptor A2 |
| INMT | 170 | -33.15 | indolethylamine N-methyltransferase |
| SCARA3 | 170 | -34.01 | scavenger receptor class A, member 3 |
| ALPL | 170 | -34.84 | alkaline phosphatase, liver/bone/kidney |
| TNFSF12 | 170 | -40.14 | tumor necrosis factor (ligand) superfamily, member 12 |
| FIBD1 | 171 | -25.2 |  |
| NEIL1 | 171 | -32.19 | nei endonuclease VIII-like 1 (E. coli) |
| GREM2 | 171 | -33.03 | gremlin 2, DAN family BMP antagonist |
| SIRPA | 171 | -33.84 | signal-regulatory protein alpha |
| IRX4 | 171 | -34.5 | iroquois homeobox 4 |
| RAPGEF3 | 171 | -34.88 | Rap guanine nucleotide exchange factor (GEF) 3 |
| TXNRD2 | 171 | -35.01 | thioredoxin reductase 2 |
| CCDC120 | 171 | -37.03 | coiled-coil domain containing 120 |
| EPAS1 | 171 | -38.36 | endothelial PAS domain protein 1 |
| GNAL | 172 | -28.98 | guanine nucleotide binding protein (G protein), alpha activating activity polypeptide, olfactory type |
| GNG2 | 172 | -30.1 | guanine nucleotide binding protein (G protein), gamma 2 |
| P2RX3 | 172 | -31.03 | purinergic receptor P2X, ligand-gated ion channel, 3 |
| KLHL29 | 172 | -31.19 | kelch-like family member 29 |
| ACVR1C | 172 | -31.84 | activin A receptor, type IC |
| ACACB | 172 | -32.16 | acetyl-CoA carboxylase beta |
| LMOD3 | 172 | -33.14 | leiomodin 3 (fetal) |
| RGL1 | 172 | -35.29 | ral guanine nucleotide dissociation stimulator-like 1 |
| KCNK17 | 172 | -37.55 | potassium channel, subfamily K, member 17 |
| LYRM4 | 172 | -40.51 | LYR motif containing 4 |
| ANGPTL4 | 173 | -28.16 | angiopoietin-like 4 |
| GRID1 | 173 | -28.17 | glutamate receptor, ionotropic, delta 1 |
| IL33 | 173 | -32.77 | interleukin 33 |
| TLN2 | 173 | -33.7 | talin 2 |
| CLEC4M | 173 | -34.43 | C-type lectin domain family 4, member M |
| ADAM11 | 173 | -36.11 | ADAM metallopeptidase domain 11 |
| TRIM2 | 174 | -31.87 | tripartite motif containing 2 |
| KCNMB1 | 174 | -32.43 | potassium large conductance calcium-activated channel, subfamily M, beta member 1 |
| SLC23A2 | 174 | -32.64 | solute carrier family 23 (ascorbic acid transporter), member 2 |
| JAM3 | 174 | -32.77 | junctional adhesion molecule 3 |
| ST8SIA2 | 174 | -33.46 | ST8 alpha-N-acetyl-neuraminide alpha-2,8-sialyltransferase 2 |
| LAMA4 | 174 | -44.11 | laminin, alpha 4 |
| FUNDC2 | 175 | -28.18 | FUN14 domain containing 2 |
| IL22RA1 | 175 | -29.87 | interleukin 22 receptor, alpha 1 |
| FOXP2 | 175 | -32.31 | forkhead box P2 |
| RPS9 | 175 | -32.95 | ribosomal protein S9 |
| PPARA | 175 | -34.38 | peroxisome proliferator-activated receptor alpha |
| RSPO1 | 175 | -36.28 | R-spondin 1 |
| CX3CL1 | 175 | -37.43 | chemokine (C-X3-C motif) ligand 1 |
| ATP13A4 | 175 | -37.44 | ATPase type 13A4 |
| PLAC9 | 176 | -31.53 | placenta-specific 9 |
| APOL4 | 176 | -32.77 | apolipoprotein L, 4 |
| FLRT2 | 176 | -33.5 | fibronectin leucine rich transmembrane protein 2 |
| HNMT | 176 | -34.85 | histamine N-methyltransferase |
| ZNF395 | 176 | -37.44 | zinc finger protein 395 |
| FLT4 | 177 | -37.13 | fms-related tyrosine kinase 4 |
| DAB2IP | 177 | -37.34 | DAB2 interacting protein |
| CCDC38 | 178 | -23.37 | coiled-coil domain containing 38 |
| CTNND1 | 178 | -31.5 | catenin (cadherin-associated protein), delta 1 |
| POM121 | 178 | -33.73 | POM121 transmembrane nucleoporin |
| ARID5A | 178 | -34.49 | AT rich interactive domain 5A (MRF1-like) |
| TMCC3 | 178 | -35.65 | transmembrane and coiled-coil domain family 3 |
| CPEB1 | 178 | -39.21 | cytoplasmic polyadenylation element binding protein 1 |
| ITSN1 | 179 | -33.29 | intersectin 1 (SH3 domain protein) |
| LIMS2 | 179 | -35.88 | LIM and senescent cell antigen-like domains 2 |
| COLEC11 | 179 | -36.21 | collectin sub-family member 11 |
| FOXO4 | 179 | -40.14 | forkhead box O4 |
| KLB | 180 | -32.29 | klotho beta |
| VPRBP | 180 | -37.68 | Vpr (HIV-1) binding protein |
| ALS2CL | 184 | -44.47 | ALS2 C-terminal like |
| SEMA3G | 186 | -31.39 | sema domain, immunoglobulin domain (Ig), short basic domain, secreted, (semaphorin) 3G |
| H6PD | 188 | -41.79 | hexose-6-phosphate dehydrogenase (glucose 1-dehydrogenase) |

**Supplementary Table S5F. List of gene targets for hsa_piR_019914**

| **Gene targets for hsa_piR_019914** | **Alignment score** | **Energy score** | **Gene name** |
| --- | --- | --- | --- |
| CXCL12 | 170 | -27.73 | chemokine (C-X-C motif) ligand 12 |
| GRHL1 | 170 | -30.36 | grainyhead-like 1 (Drosophila) |
| PLCD3 | 170 | -34.65 | phospholipase C, delta 3 |
| MFI2 | 170 | -35.68 | antigen p97 (melanoma associated) identified by monoclonal antibodies 133.2 and 96.5 |
| PDE2A | 170 | -37.29 | phosphodiesterase 2A, cGMP-stimulated |
| TMED4 | 171 | -34.89 | transmembrane emp24 protein transport domain containing 4 |
| SEC63 | 171 | -35.44 | SEC63 homolog (S. cerevisiae) |
| XPNPEP3 | 171 | -35.44 | X-prolyl aminopeptidase (aminopeptidase P) 3, putative |
| TXNRD2 | 171 | -36.14 | thioredoxin reductase 2 |
| SLC6A14 | 171 | -36.16 | solute carrier family 6 (amino acid transporter), member 14 |
| KIF1B | 171 | -36.73 | kinesin family member 1B |
| ANKDD1A | 171 | -37.17 | ankyrin repeat and death domain containing 1A |
| GHR | 171 | -37.39 | growth hormone receptor |
| LEPR | 172 | -32.43 | leptin receptor |
| ITM2C | 172 | -38.62 | integral membrane protein 2C |
| RRAD | 172 | -40.81 | Ras-related associated with diabetes |
| EPB41L4B | 172 | -42.09 | erythrocyte membrane protein band 4.1 like 4B |
| ZNF506 | 173 | -30.73 | zinc finger protein 506 |
| DAB2IP | 173 | -43.79 | DAB2 interacting protein |
| ATP1B2 | 174 | -38.89 | ATPase, Na+/K+ transporting, beta 2 polypeptide |
| CDC14B | 175 | -37.63 | cell division cycle 14B |
| CA5B | 175 | -37.66 | carbonic anhydrase VB, mitochondrial |
| HSD17B13 | 175 | -37.66 | hydroxysteroid (17-beta) dehydrogenase 13 |
| IGF1 | 175 | -37.66 | insulin-like growth factor 1 (somatomedin C) |
| IL17RD | 175 | -37.66 | interleukin 17 receptor D |
| LY75 | 175 | -37.66 | lymphocyte antigen 75 |
| LYRM4 | 175 | -37.66 | LYR motif containing 4 |
| PER2 | 175 | -37.66 | period circadian clock 2 |
| PHLDA1 | 175 | -37.66 | pleckstrin homology-like domain, family A, member 1 |
| PYGO1 | 175 | -37.66 | pygopus family PHD finger 1 |
| PTCHD1 | 175 | -37.66 | patched domain containing 1 |
| PSTPIP2 | 175 | -37.66 | proline-serine-threonine phosphatase interacting protein 2 |
| SYNPO2 | 175 | -37.66 | synaptopodin 2 |
| EREG | 175 | -38.02 | epiregulin |
| ALG9 | 175 | -38.15 | ALG9, alpha-1,2-mannosyltransferase |
| ANTXR2 | 175 | -38.23 | anthrax toxin receptor 2 |
| ZC3H6 | 175 | -38.58 | zinc finger CCCH-type containing 6 |
| ARRB1 | 176 | -36.37 | arrestin, beta 1 |
| KCNH8 | 177 | -26.99 | potassium voltage-gated channel, subfamily H (eag-related), member 8 |
| ACO1 | 177 | -43.07 | aconitase 1, soluble |
| NARG2 | 179 | -38.72 | NMDA receptor regulated 2 |
| PTPN3 | 179 | -39.29 | protein tyrosine phosphatase, non-receptor type 3 |
| EHD2 | 179 | -42.58 | EH-domain containing 2 |
| B3GAT1 | 180 | -37.43 | beta-1,3-glucuronyltransferase 1 (glucuronosyltransferase P) |
| CLPX | 183 | -42.5 | caseinolytic mitochondrial matrix peptidase chaperone subunit |
